# The costs and cost effectiveness of providing first-trimester, medical and surgical safe abortion services in KwaZulu-Natal Province, South Africa

**DOI:** 10.1371/journal.pone.0174615

**Published:** 2017-04-03

**Authors:** Naomi Lince-Deroche, Tamara Fetters, Edina Sinanovic, Jaymala Devjee, Jack Moodley, Kelly Blanchard

**Affiliations:** 1 Ibis Reproductive Health, Johannesburg, South Africa; 2 Ipas, Chapel Hill, NC, United States of America; 3 Health Economics Unit, School of Public Health and Family Medicine, University of Cape Town, Cape Town, South Africa; 4 King Dinuzulu Hospital, Department of Obstetrics and Gynaecology, Durban, South Africa; 5 Nelson R Mandela School of Medicine, University of KwaZulu-Natal, Durban, South Africa; 6 Ibis Reproductive Health, Cambridge, MA, United States; University of Ottawa, CANADA

## Abstract

**Background:**

Despite a liberal abortion law, access to safe abortion services in South Africa is challenging for many women. Medication abortion was introduced in 2013, but its reach remains limited. We aimed to estimate the costs and cost effectiveness of providing first-trimester medication abortion and manual vacuum aspiration (MVA) services to inform planning for first-trimester service provision in South Africa and similar settings.

**Methods:**

We obtained data on service provision and outcomes from an operations research study where medication abortion was introduced alongside existing MVA services in public hospitals in KwaZulu-Natal province. Clinical data were collected through interviews with first-trimester abortion clients and summaries completed by nurses performing the procedures. In parallel, we performed micro-costing at three of the study hospitals. Using a model built in Excel, we estimated the average cost per medical and surgical procedure and determined the cost per complete abortion performed. Results are presented in 2015 US dollars.

**Results:**

A total of 1,129 women were eligible for a first trimester abortion at the three study sites. The majority (886, 78.5%) were eligible to choose their abortion procedure; 94.1% (n = 834) chose medication abortion. The total average cost per medication abortion was $63.91 (52.32–75.51). The total average cost per MVA was higher at $69.60 (52.62–86.57); though the cost ranges for the two procedures overlapped. Given average costs, the cost per complete medication abortion was lower than the cost per complete MVA despite three (0.4%) medication abortion women being hospitalized and two (0.3%) having ongoing pregnancies at study exit. Personnel costs were the largest component of the total average cost of both abortion methods.

**Conclusion:**

This analysis supports the scale-up of medication abortion alongside existing MVA services in South Africa. Women can be offered a choice of methods, including medication abortion with MVA as a back-up, without increasing costs.

## Introduction

Achieving universal access to sexual and reproductive health services was a target under the Millennium Development Goals (MDGs) [1] and is part of the newly ratified Sustainable Development Goals (SDGs) framework [2]. Attempts to estimate the resources required to meet the MDGs included the costs of scaling up access to maternal health services and the costs of meeting unmet need for contraception [3–7]. Abortion-related costs have also been addressed, but generally the costs included have been those associated with unsafe abortion, i.e. costs to be averted through increases in contraception or reductions in unwanted pregnancy. This is important, given the magnitude of unsafe abortion globally and its associated costs [8]. However, not including the costs of providing safe abortion services is a limitation considering that most women live in countries where abortion is legally permitted under a range of circumstances, and almost every country permits abortion under some circumstances [9]. Further, even where contraceptive prevalence is high, contraceptive failure or non-use is a reality.

Efforts are underway to estimate the investment requirements for attaining the new SDGs [10]. South Africa in particular is exploring the potential for meeting its commitments on sexual and reproductive health [11]. Since 1997, abortion has been legal in the first trimester without restriction, and between 13–20 weeks gestation it is legal for cases of socio-economic hardship, rape, incest and for reasons related to the health of the pregnant woman or fetus [12]. Despite its liberal law, however, access to abortion services in South Africa is challenging for many women for a host of reasons including a lack of willing providers and stigma [13–15]. It has been suggested that introduction of medication abortion has the potential to improve access in the country [16]. Mifepristone was approved for use in 2001 [16], but medication abortion was only introduced in the public sector in 2013, and is still not available in some provinces, in part due to concerns over the high cost of the medication. An operations research study by Blanchard et al (2015) [17] explored the impact of introducing medication abortion—with a combined mifepristone-misoprostol regimen—alongside existing first-trimester manual vacuum aspiration (MVA) services in four public hospitals in KwaZulu-Natal province. The majority of eligible patients chose to have a medical procedure, and the service was shown to be both safe and acceptable. In this study, we estimate the costs and cost-effectiveness of providing legal first-trimester medication abortion and MVA services in the study by Blanchard et al. to inform planning for first-trimester service provision in South Africa and similar settings.

## Materials and methods

### Clinical services and effectiveness outcomes

We derive the service information and clinical outcomes for this economic evaluation from the operations research study conducted by Blanchard et al [17]. Approval for the operations research study and this cost evaluation was received from the University of KwaZulu-Natal’s Biomedical Research Ethics Committee, Allendale Institutional Review Board, the KwaZulu-Natal Department of Health, and the study facilities. Women participating in the operations research study provided written informed consent.

From 2009 to 2011 women who were ≤9 weeks gestation, clinically eligible for medication abortion, and enrolled in the operations research study were able to choose either medication abortion or MVA. Women who were between 10 and 12 weeks gestation were also invited to enroll, and had the standard MVA service because they were not eligible for medication abortion due to advanced gestational age. This configuration for service delivery in the study where women ≤9 weeks gestation chose their procedure and women 10–12 weeks gestation all had MVA reflects the expected standard of care where both MVA and medication abortion are offered in South Africa.

KwaZulu-Natal’s provincial guidelines for all first-trimester abortion services advise a comprehensive work up with testing for anemia and syphilis [18]. Ultrasound for gestational age dating is recommended but not required [18]. The standard of care for MVA (for women ≤12 weeks gestation) across South Africa includes a visit for the initial work up and counseling and a second visit for the procedure and contraception. No follow-up visit is required after MVA.

National guidelines for medication abortion indicate that women should take mifepristone (200 milligrams) at their first visit to the facility and receive misoprostol (800 micrograms) to take at home 48 hours later [19]. Completion of the medication abortion is assessed through questions and abdominal palpitations at a routinely scheduled follow-up visit, where ultrasound is advised but not required for confirmation of completion [19].

In the operations research study, at each woman’s first visit to the facility, study staff conducted an interview (S1 Form), and the facility nurse documented clinical procedures (S2 Form). All women who were eligible to choose their procedure (i.e. those ≤9 weeks gestation) were invited to return for a study follow-up interview 10 to 21 days later. At the study follow-up visit, the procedure outcome and other clinical and acceptability data were collected by the facility nurse. Also during the study, women who had chosen their procedure may have attended the study facilities for an “unscheduled visit” before or after the study’s required follow-up visit. If the woman had an unscheduled visit to the facility, a form was completed by study staff indicating the reasons for the visit and treatment provided or action taken if any.

Women who enrolled in the study but were not eligible to choose their abortion procedure because they were not eligible for medication abortion had one initial interview only–prior to their procedure. They were not asked to return for a follow-up study visit after their MVA, and unscheduled visits were also not recorded for them if such visits occurred.

The possible outcomes for all women undergoing abortion in the operations research study included having 1) a complete, uncomplicated abortion; 2) a failed abortion procedure without complications; 3) a failed abortion procedure with complications, or 4) an ongoing pregnancy at the time of exiting the study. A “failed” procedure was defined per the study protocol as needing a (repeat) MVA post-procedure as a result of an incomplete abortion or prolonged/severe bleeding or discomfort. As noted above, procedural outcomes were documented at the study follow-up visit. For the purposes of this analysis, women who did not return for follow-up were assumed to have had a complete, uncomplicated abortion. We justify this assumption based on the similarity of our overall success rate (including the assumed successes) to published literature [20]; however, we also test this assumption in sensitivity analysis (see below).

Finally, if the forms completed during the operations research study did not cover clinical information required for the cost evaluation and the data were unknown by the nurses at any facility, we used national data reported in South African literature. This was done specifically for anemia prevalence, bacterial sexually transmitted infection (STI) prevalence, and Rhesus factor information [21–24]. These data were then used to determine the costs for treating anemia, bacterial STIs and Rho(D)-immune globulin injections at all sites.

### Costing

We conducted a bottom-up, or micro, cost evaluation from the health service perspective at three of the operations research study facilities throughout 2011–2013. One very low-volume facility was not included due to logistically difficulties. Generally a “bottom-up” evaluation involves first determining all resources used and then multiplying the resource usage, or volume, by the cost per resource type to obtain a total cost. For this evaluation, staff, medication, laboratory, consumables, and equipment costs were included. This included resources used for all activities, e.g. testing at the intake visit, the actual procedure, medications given for conditions identified, etc. To obtain resource utilization data, including the staff time per procedure, we interviewed the staff involved in providing abortion services at each site, asking them to describe their standard practice. Then we used publicly available sources, facility expenditure records and information from medical suppliers to determine the unit costs for laboratory tests, personnel, medications, consumables and equipment [25–27]. We assume the full costs of equipment apply to abortion services, i.e. that equipment is not shared across other services, because it is common in South Africa for abortion services to be segregated from other obstetric and gynecological services. We annualized equipment costs using a discount rate of 3% and depreciation periods recommended by the South Africa Revenue Service [28], and then divided the annual, depreciated costs by annual service volume to obtain a cost per service. All costs were collected in South African Rand, inflated to 2015 prices [29], and are reported here in 2015 US dollars using an average exchange rate for 2015 of 14.39 Rands per dollar [30].

Costs for complications were estimated in two ways. For women who experienced a failed initial procedure–MVA or medication abortion–and who subsequently required a follow-up MVA procedure and for which no other complication (i.e. hemorrhage, sepsis, shock, laceration or perforation) was present, we assumed the cost of the follow-up MVA was the same as the total cost of an initial MVA to induce abortion. For women in the study who required overnight hospitalization for their follow-up MVA, we assumed the cost to the health system was equivalent to the published charge for a consultation with a doctor and 24-hour hospitalization (in a public facility) with a surgical procedure performed by a gynecologist with assistance from a nurse [31]. This charge includes medications and consumables as would be required [32].

The costs of complications requiring more than an evacuation as treatment (i.e. cases with hemorrhage, sepsis, shock, laceration or perforation) were explored in a sensitivity analysis as they did not occur during the operations research study. To estimate of the costs of these complications, we again used the published 24-hour hospitalization charges for a minor surgical procedure performed by a gynecologist assisted by a nurse. This estimate was based on a systematic review of approaches and costs for treating post-abortion care globally that showed that the weighted mean length of hospitalization for care was 22.6 hours [33].

With the exception of hospitalization costs, the costs estimated and presented for each procedure are incremental in that they do not include infrastructural costs such as rent, utilities and some personnel (e.g. security guards, kitchen staff, etc.). These costs are excluded due to the complexity of estimating them in a hospital setting, the potential for wide variation from facility to facility, and to allow for comparability with similar cost evaluations from other settings (which exclude overhead) [34–39]. We also excluded training costs (because at the time of the study, abortion training was provided by an NGO), the costs of contraceptive commodities and contraceptive counseling not offered as part of routine abortion counseling (because those are budgeted for separately), and women’s costs for accessing services.

### Analysis

Clinical data from the operations research study were analyzed in Stata (Release 14. College Station, TX: StataCorp LP). From the clinical dataset we established service volume, ultrasound usage rates, eligibility for medication abortion, abortion method choice among eligible women, procedure outcomes, the proportion of women who received analgesics, hospitalization rates, and follow-up visit attendance. We also evaluated the proportion of women who had an unscheduled visit and services provided at unscheduled visits.

**Fig 1:**
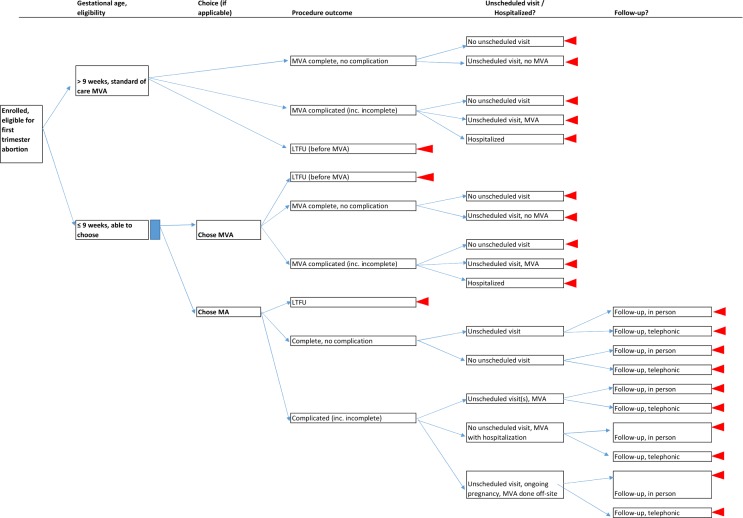
Decision tree for medication abortion and MVA service outcomes, based on operations research study [17]. MA = medication abortion, MVA = manual vacuum aspiration, LTFU = lost to follow up NB: Unscheduled visits among MA women may have occurred before or after the scheduled follow-up visit. For simplicity, all are shown here before the follow-up visit.

We captured and analyzed cost data by study site using a model built in Microsoft Excel (2013). The model contents are classified by category: 1) source data and unit costs, 2) research usage data collected through micro-costing activities and analysis of the operations study database, and 3) analytical work and outcomes (see the full listing of contents in S1 Workbook snapshot). Within the model, we established a decision tree representing all possible visit schedules and procedure outcomes (Fig 1), and inputted operations research study outcomes and average costs per patient interaction (e.g. first visit, MVA procedure visit, unscheduled visit, hospitalization, in-person follow-up, telephonic follow-up, etc.) to establish the average total cost per procedure. Because a follow-up visit is not standard of care for MVA services in South Africa, we did not include the costs of study follow-up visits for those women in the average procedural cost calculations or in the decision tree analysis (Fig 1).

We present the total average cost per procedure with a breakdown of costs (i.e. personnel, medications, laboratory tests, consumables, equipment, and hospitalization (if applicable)). We also show the average costs for complicated and uncomplicated procedures and provide an indication of their contribution to the overall total average costs.

For the cost effectiveness evaluation, the outcome is defined as the cost per complete abortion. We estimate the total cost of all abortion services in the study–complicated and uncomplicated–and divide that by the number of complete abortions at the time of study exit. Complete abortions are defined as any procedure or combination of procedures that successfully terminated the pregnancy, so, for example, a failed medication abortion followed by a successful MVA was considered to be a complete abortion.

To address uncertainty in the model, we varied personnel time, supply and equipment costs and hospitalization costs by ± 25% to create a range around each base cost value (See S1 Workbook snapshot). Costs for staff (i.e. monthly salaries), medication and laboratory tests were not varied because these costs are published publicly in South Africa on a routine basis. Hospitalization costs are also published; however, there is some variation in how those rates are applied from facility to facility.

We also conducted univariate and bivariate sensitivity analysis to explore the impact of variations in model inputs on the cost and cost-effectiveness outcomes (See S1 Workbook snapshot). Because complications for both medication abortion and MVA are rare and the operations research study by Blanchard et al. [17] was not a clinical trial powered to assess differences in completion or complication rates between the procedures, we reviewed the literature to assess possible ranges for completion and complications. The impact of loss-to-follow-up in the study was also explored.

## Results

During the operations research study, a total of 1,129 women presented at the three study facilities where costing took place and were eligible for a first trimester abortion (Table 1). The majority (886, 78.5%) were eligible to choose their abortion procedure type; 94.1% (n = 834) chose medication abortion. Eighty-six percent (n = 714) of medication abortion clients returned for their scheduled follow-up visit.

**Table 1:** Service volume, procedure visits, and outcomes from three sites in operations research study (N = 1,129)* [17].

	Number (%) unless otherwise indicated
Abortion services provided monthly (study only) (median [IQR])	18 [12–34]
Total abortion services offered in study (n)	1,129
Had standard of care MVA (not eligible to choose)	243 (21.5)
Eligible to choose procedure	886 (78.5)
Chose medication abortion	834 (94.1)
Outcomes for women who had MVA at ≤ 9 weeks (n = 52)	
Lost-to-follow-up	19 (36.5)
Returned for required study follow-up	33 (63.5)
Complete/uncomplicated procedure	33 (100.0)
Failed procedure, required repeat MVA as outpatient service	0 (0.0)
Failed procedure, hospitalized for completion/management	0 (0.0)
Failed procedure, ongoing at study exit	0 (0.0)
Outcomes for women who had MA at ≤ 9 weeks (n = 834)	
Lost-to-follow-up	120 (14.4)
Returned for required study follow-up	714 (85.6)
Complete/uncomplicated procedure	691 (96.8)
Failed procedure, required MVA as outpatient service	18 (2.5)
Failed procedure, hospitalized for completion/management	3 (0.4)
Failed procedure, ongoing at study exit**	2 (0.3)
Unscheduled visits among medication abortion clients (n = 31 visits)***	
Saw the study nurse	30 (97.0)
Examined by nurse	9 (29.0)
Received extra counselling from nurse	5 (16.1)
Ultrasound	1 (3.2)
Given more misoprostol	7 (22.6)
Given second dose of mifepristone due to vomiting of first dose	1 (3.2)
MVA scheduled	9 (29.0)
MVA performed	18 (58.1)
Given antibiotics	1 (3.2)
Given pain medication	1 (3.2)
Given anti-emetic medication	1 (3.2)
Referred to other service	1 (3.2)

MA = medication abortion.

* Four sites participated in the operations research study. Costing for this evaluation occurred at only three.

** These women reported that the MVA was performed elsewhere after study exit.

*** Twenty-five medication abortion clients had 31 unscheduled visits; more than one service may have been provided per visit.

Considering the women who chose medication abortion and returned for a study follow-up visit (n = 714), 96.8% (n = 691) had a complete medication abortion. Twenty-three women were considered to have had a failed medication abortion: 18 had an MVA at a study facility; three women were hospitalized overnight; and two women had ongoing pregnancies at study follow-up but later reported receiving an MVA outside of the study facility.

Twenty-five women in the medication abortion group (3.0%, 25/834) had an “unscheduled” visit, i.e. a visit that was in addition to the initial presentation or scheduled study follow-up visit. Procedures or treatment provided at unscheduled visits are shown in Table 2. Seeing a nurse and having an MVA performed or scheduled were the most common services obtained during unscheduled visits.

**Table 2:** Resources required* for safe medication abortion and MVA procedures.

Category	Resources	
*For both procedures*:		
Personnel**	Nurse midwife, staff nurse, ultrasound technician (if ultrasound done)	
Consumables	Office supplies, hand washing/sanitizing supplies, ultrasound gel (if done), supplies for exam and urine/blood testing	
Medication	Analgesics, misoprostol, iron tablets (if given)	
Equipment	Waiting room furnishings, consulting room furnishings, ultrasound machine (if used), equipment for assessing vital signs/rapid blood tests	
Laboratory	Blood sent to national lab for syphilis testing (if done)	
*Additional resources*:	*MA only*	MVA only***
Personnel	None	Medical specialist for MVA after MA if hospitalized
Consumables	Phone call cost if needed for follow-up	MVA cannulas, gloves, masks, linen savers, cotton swabs, sanitary towels, etc.
Medication	Mifepristone	Antibiotics (if given)
Equipment	None	MVA aspirator**** and other small medical equipment, theater/operating room furnishings, recovery room furnishings

MA = medication abortion, MVA = manual vacuum aspiration.

*Some sites did not use all of the resources noted here. Some resources were systematically not used (e.g. Rhesus testing at one site), and some resources were occasionally not used (e.g. ultrasound when not available or clinically indicated).

**To aid comparability with other countries, training for nurses in South Africa is as follows: Staff nurse—2 years, Nurse midwife—6 years.

***MVA if chosen by the woman or if needed after an incomplete MA.

****According to site nursing staff, the MVA aspirators were replaced approximately every month; cannulas are replaced after each procedure.

### Resource utilization and service parameters

The resources used to provide first-trimester safe abortion procedures at the facilities are provided in Table 2. The initial abortion procedures were performed by nurse midwives.

Table 3 provides clinical parameters for procedures as conducted at each study site and for the study population as a whole. Urine-based pregnancy testing was done for less than half of the study participants; the nurses reported that women were often expected to come with the result of a pregnancy test done elsewhere. For ultrasound, the nurses reported that for some cases it was not necessary, but that at times ultrasound use was limited by technicians who refused to see more than a limited number of abortion clients per day. Some sites systematically did not do hemoglobin and blood pressure testing due to broken machinery or lack of time. Syphilis testing was not done at any site. Interestingly Rhesus testing and treatment were done despite this not being required for women under 12 weeks of gestation per the province’s abortion guidelines [18].

**Table 3:** Clinical and service parameters for safe medication abortion and MVA procedures (% unless otherwise indicated).

	Site 1 (n = 432)		Site 2 (n = 537)		Site 3 (n = 160)		All sites (n = 1,129)	
Intake visit								
Urine-based pregnancy test*	0.0		100.0		0.0		47.6	
Ultrasound for dating	96.7		32.7		89.7		65.3	
Blood pressure and temperature	3.0		100.0		0.0		48.8	
Hemoglobin test*	10.0		3.0		0.0		5.0	
Syphilis test*	0.0		0.0		0.0		0.0	
Rhesus test*	100.0		0.0		100.0		52.4	
Follow-up visit (MA clients only)								
Hemoglobin test*	0.0		0.0		100.0		14.2	
Ultrasound to assess completion	3.1		1.1		2.9		2.1	
Medications								
Iron tablets provided**	0.0		100.0		0.0		47.6	
Rh_o_(D) immune globulin*^,^ ***	15.0		0.0		15.0		7.9	
Antibiotics for STI symptoms*^,^ ***	14.9		40.0		11.9		26.8	
	*MA*	*MVA*	*MA*	*MVA*	*MA*	*MVA*	*MA*	*MVA*
Extra misoprostol*^,^ ****	1.2	10.0	0.0	1.3	1.3	10.0	0.6	5.9
Oral analgesics (paracetamol and/or NSAID)	1.9	100.0	1.9	100.0	23.2	0.0	4.9	85.8
IM analgesic injection pre-procedure (NSAID)	N/A	100.0	N/A	0.6	N/A	100.0	N/A	52.7
Prophylactic antibiotics*	0.0	0.0	0.0	60.0	0.0	0.0	0.0	28.5

MA = medication abortion, MVA = manual vacuum aspiration, N/A = Not applicable, IM = intramuscular, NSAID = nonsteroidal anti-inflammatory drug.

* Rates are as reported by the facility nurses.

** At one site, tablets were given routinely to all women post-abortion (regardless of hemoglobin levels).

*** The nurses at sites 1 and 3 reported providing treatment when indicated. However, the prevalence of Rhesus negativity and STI symptoms were unknown, so prevalence data was sourced from published literature [23,24].

**** For MA = given extra misoprostol (on average 5.25 x 200 micrograms) at an unscheduled visit due to suspected MA failure, told to return for follow-up. For MVA = given more misoprostol for dilation in the morning prior to the MVA procedure.

At two sites, all MVA patients received an injection of a nonsteroidal anti-inflammatory drug (NSAID) for pain just prior to the procedure; at the third site all women received oral NSAIDs prior to the MVA procedure. Less than 5% of all medication abortion clients received oral pain medication. The nurses reported that ibuprofen was not always available for dispensing, and that public sector clients (attending for various procedures) were frequently asked to purchase their medications elsewhere.

### Cost and cost-effectiveness

Table 4 indicates the total average costs per abortion procedure provided in the operations research study. These costs include hospitalization and unscheduled visit costs, taking into account the proportion of women who experienced those costs (as noted in Table 1). The costs for personnel are shown in total and separately for the initial procedure and for hospitalization.

**Table 4:** Total average cost and cost breakdown for provision of first-trimester medication abortion and MVA in three hospitals in KwaZulu-Natal, South Africa (USD 2015)*.

	Medication abortion (n = 834)		MVA (n = 295)		Both procedures (n = 1,129)	
	Base (Range)**	% of total	Base (Range)**	% of total	Base (Range)**	% of total
Personnel	29.79 (22.38–37.20)	46.6	39.22 (29.41–49.02)	56.3	32.25 (24.22–40.29)	49.3
Initial procedure***	29.65		39.22		32.15	
Hospitalization staff****	0.14		0.00		0.11	
Consumables	10.53 (7.90–13.16)	16.5	19.26 (14.44–24.07)	27.7	12.81 (9.61–16.01)	19.6
Medication	17.34	27.1	1.71	2.5	13.26	20.3
Equipment	5.30 (3.98–6.61)	8.3	9.41 (7.06–11.77)	13.5	6.37 (4.79–7.96)	9.7
Laboratory	0.00	0.0	0.00	0.0	0.00	0.0
Hospitalization, other****	0.95 (0.71–1.19)	1.5	0.00 (0.00–0.00)	0.0	0.70 (0.53–0.88)	1.1
**Average total cost**	**63.91 (52.32–75.51)**	**100.0**	**69.60 (52.62–86.57)**	**100**	**65.40 (52.40–78.40)**	**100.0**

* Costs take into consideration the proportion of women with failed abortions and (repeat) MVAs to complete the procedure, complication/hospitalization rates, unscheduled visit rates, and study follow-up visit rates as presented in Table 1. All costs are incremental except for hospital costs which represent published charges and include overhead.

** Ranges in parentheses represent ±25% changes in personnel time, supply and equipment costs and hospitalization costs.

*** This includes a first visit, performing the MVA procedure if required, unscheduled visits, study follow-up visits for medication abortion women, and any administrative “extra” time required for inventory, paperwork, etc.

**** “Hospitalization, staff” refers to the doctor performing the consultation, the gynecologist performing a (repeat) MVA, and the nurse who assists the gynecologist. “Hospitalization, other” refers to the “hotel” cost associated with staying for 24 hours and the non-personnel costs of having a procedure performed in an operating theater.

The ranges presented in Table 4 show variation in the estimated costs based on uncertainty analysis conducted for the cost inputs (i.e. varying personnel time, supply and equipment costs, and hospitalization costs by ±25%). Considering the base case cost estimates, medication abortion was less costly than MVA. However, considering the uncertainty analysis ranges, the plausible ranges for the costs of the two procedures overlap, suggesting that the costs of the two procedures could be the same in some circumstances. This is explored further in the sensitivity analysis (see below).

Personnel costs were the largest contributor to total costs for both procedures. Given the extremely low rate of hospitalization among women who had a medical abortion and a study follow-up visit (0.4%, 3/714)) in the operations research study, the contribution of hospitalization costs to the total average cost of medication abortion is minimal. Because no women who had an MVA were hospitalized, there are no hospitalization costs for MVA. Laboratory costs are zero for both procedures because no site conducted investigations requiring outsourced testing.

Table 5 provides the total cost of abortions performed during the operations research study as well as the cost effectiveness outcomes. More was spent on medication abortion procedures than MVA procedures simply because more women had medication abortion procedures. The average cost for a complete/uncomplicated medication abortion was $61.06, and the average cost for a complete/uncomplicated MVA was $69.60. For a failed procedure with hospitalization for complications, the cost per complete medication abortion was $164.47. The cost per complete abortion was $64.06 for medication abortion and $69.60 for MVA. The total costs to the health service of providing 1,129 first-trimester abortions during the 16-month study period was $73,833.

**Table 5:** Average cost per outcome, total costs (USD 2015), and proportion attributable to complete versus incomplete first-trimester medication abortion and MVA in three hospitals in KwaZulu-Natal, South Africa*.

	Medication abortion (n = 834)	MVA* (n = 295)	Both procedures (n = 1,129)
	**$**	**$**	**$**
Total cost during study	53,299	20,351	73,833
Average cost per…			
Complete/uncomplicated procedure	61.06	69.60	63.33
Failed and/or complicated** procedure	164.47	N/A	164.47
Complete abortion***	64.06	69.60	65.51
	**%**	**%**	**%**
Percent of total costs attributable to…			
Complete/uncomplicated procedures	92.9	100.0	94.9
Failed/complicated procedures	7.1	0.0	5.1

MVA = manual vacuum aspiration.

* These women had and MVA either because they were not eligible to choose or because they chose MVA over medication abortion.

** In the operations research study, complications were deemed to be those conditions requiring hospitalization.

*** The denominator for medication abortion excludes the two women with ongoing pregnancies at study exit. For both medication abortion and MVA, all women who were lost to follow-up were assumed to have had a complete abortion.

### Sensitivity analysis

Table 6 provides further detail on the model inputs and ranges explored in the sensitivity analysis. The total average cost of medication abortion was moderately sensitive to the cost of mifepristone. Reducing the price of mifepristone by a hypothetical 50% per 200 mg tablet to a price of $8.00 (113.76 South African Rands) would result in a 14.1% decrease in the average cost per complete medication abortion, resulting in a cost of $56.13 per complete abortion. In contrast, procedural costs were relatively insensitive to the lifespan of the MVA aspirator; reducing its lifespan from the reported 30 days to an average of 7 days would result in only minimal increases in the average cost of a complete medication abortion (by 0.1%) and MVA (by 4.0%).

**Table 6:** Uncertainty and sensitivity analysis base case parameters and ranges.

Parameter	Base	Range	Source for range
*Uncertainty analysis for base case ranges*			
Personnel time	Varied by procedure*	±25%	—
Total costs for supplies and equipment	See Table 4.	±25%	—
Hospitalization cost for 24 hour stay	$195**	±25%	—
*Sensitivity analysis*			
Depreciation rate	3%	3%, 5%	—
Mifepristone cost (per 200 mg)	$16	$8–16	—
MVA aspirator lifespan	30 days	7–30	—
Proportion of women eligible for MA	78.5%	50–78.5	—
Proportion of eligible women who chose MA	94.1%	50–94.1	—
Completion rates, among those with follow-up***			
MA	96.8%	95–98	[20,40]
MVA	100.0%	95–100	[20,41]
Complication (hospitalization) rate, among those with follow-up (excl. failures)****			
MA	0.4%	0.0–5.0	[41,42]
MVA	0.0%	0.0–5.0	[41]
Follow-up visit rate ^*****^			
MA	85.6%	0–100	—
MVA	0.0%	0–10	—

MA = medication abortion, MVA = manual vacuum aspiration, $ = 2015 US dollars, excl. = excluding

* For the main provider, i.e. the nurse midwife, the estimated time required per medication abortion was 94 minutes; per MVA it was 87 minutes.

** The base case estimate includes a consultation with a doctor, one night of hospitalization, operating theater costs for a surgical procedure, and costs for the specialist and nursing staff who perform the procedure [31].

*** In the operations research study, 37 of 52 MVA women returned for follow-up. All had a complete abortion. It was assumed that women who were lost to follow-up also had complete abortions. For medication abortion women, it was assumed that women without follow-up also had a complete abortion.

**** We have rounded both upper limits to 5.0% for comparability. In the report by Niinimaki et al [41], 21.1% of women undergoing medication abortion had “complications” however, 15.6% were “reported hemorrhage.” The remaining 5.5% had other complications (e.g. hemorrhage with evacuation, sepsis, lacerations, etc.). For MVA, Niinimaki et al [41] reported a complication rate of 5.98%.

^*****^ For medication abortion, when follow-up occurs, a constant ratio of in-person (81.7%) to telephonic (18.3%) visits is assumed. For MVA, the 0% reflects what was required according to the standard of care and what was included in the costing; the 0–10% range reflects what was tested in terms of additional costs if some women were to return for follow-up.

Questions in South Africa regarding the proportion of women presenting for abortion who are eligible for medication abortion and, of those, the proportion who might choose medication abortion prompted exploration of the impact of varying the rates observed in the study. Simultaneously reducing both medication abortion eligibility and choice to 50% resulted in a 0.7% increase in complete medication abortion costs due to the lowered service volume.

Published literature suggests that the completion rate for medication abortion may vary from 95–98% [20,40]. For this analysis, reducing the completion rate for medication abortion to 95% and holding the hospitalization rate constant at 0.4% increased the total average cost per complete procedure by 1.8%. However, simultaneously reducing the completion rate to 95% and increasing the hospitalization/complication rate to 5% increased the cost per complete medication abortion by 13.6% to $74.13. Reducing the completion rate for MVA to 95% (based on published completion rates [20,41]) and simultaneously assuming a hospitalization/complication rate of 5% resulted in 18.0% increase in the cost per complete procedure to $84.83.

Finally, technical guidance from the World Health Organization (WHO) suggests that routine in-person follow-up after medication abortion may not be required and that a two-week postoperative check-in after MVA may be advisable for a minority of women [20]. Increasing the proportion of women who undergo a medication abortion and return for follow-up from the observed 85.6% to 100% increased the cost per complete procedure to $65.83; in contrast, reducing the follow-up visit rate to 0%, reduced the cost by 19.4% to $53.66. For MVA, increasing the follow-up visit rate from 0% to a hypothetical 10% resulted in a 1.4% increase in cost to $70.60 per complete abortion.

## Discussion

In an operations research study where medication abortion was introduced alongside existing MVA services, the estimated total average cost per complete medication abortion was lower than the cost estimated for MVA; however, uncertainty analysis yielded overlapping ranges.

The operations research study was not designed to assess differences in completion or complication rates, and due to the rarity of complications with safe, first-trimester services, no complications were observed for MVA services in the study. As a result, the rare possibility of complications was addressed in this evaluation through sensitivity analysis. Lower completion rates and simultaneously higher complication rates would increase the cost per complete medication abortion and MVA. However comparing the worst case scenario (i.e. lowest completion rates and highest complication/hospitalization rates) for both procedures, medication abortion remained less costly than MVA.

In the operations research study conducted by Blanchard et al [17], women preferred medication abortion over MVA, and thus the total health service cost during the study (nearly $74,000) largely reflected the lower cost of medication abortions. There have been discussions regarding the relatively high cost of mifepristone globally [43,44]. In this analysis, although drug costs contributed 27% of the total cost of medication abortion (in part because mifepristone cost $16 per tablet), the very low cost of equipment and consumables meant that the procedure was still competitive with MVA from a cost perspective. Efforts to reduce the cost of mifepristone would further decrease the average and total costs of medication abortion.

The costs reported here reflect a bottom-up approach to cost evaluation, meaning that they may not reflect the costs of these services if provided according to national or provincial guidelines. In fact, the facility nurses reported challenges with following guidelines for abortion provision as a result of stigma from ultrasound service providers in the facilities, a lack of resources, and poor maintenance of hospital equipment.

A limited number of studies have estimated the costs of offering legal abortion in low- or middle-income settings. In 2009, Hu et al. [36] reported that the direct medical costs in Mexico City for hospital-based MVA and clinic-based medication abortion (misoprostol only) were (USD 2005) $107 and $69 respectively. In 2010, Hu et al. [35] estimated that the direct medical cost of hospital-based MVA was (USD 2007) $33.11 in Nigeria and $14.58 in Ghana. The same study indicated that clinic-based medication abortion (misoprostol alone) cost $16.40 in Nigeria and $4.17 in Ghana. In both papers, Hu et al discussed the importance of the cadre of health care worker performing the procedure in different kinds of facilities as an important factor in determining the cost outcomes. In South Africa, nurses provide first-trimester procedures whether they are performed in a hospital or clinic, and personnel costs were the largest component of average procedural costs in this study.

Additional studies have reported on the costs of medical and surgical approaches for postabortion care. Vlassoff et al (2012) reported that the costs of managing postabortion complications in Ethiopia ranged from (US 2008) $23.69 for an evacuation with MVA to $153.15 for management of uterine perforations. The average cost for managing any postabortion complication was $30.69 [45]. In 2015, Vlassoff et al reported that the direct medical cost of managing any postabortion complication in Rwanda was (USD 2010) $47.05 [46]. For this study, we assumed that the cost of managing any complication was equivalent to the published cost of (USD 2015) $195 for overnight hospitalization with a gynecological procedure performed in an operating theater. This was likely an overestimate but with minimal impact in this analysis due to low complication and hospitalization rates.

This analysis has limitations. The data are drawn from a study conducted in one province only. Although 1,192 women participated at the three hospitals where costing took place, we did not observe incomplete or complicated MVA procedures, which are generally very rare [20]. Complications with medication abortion were also rare in the study. Loss to follow-up may have resulted in missing documentation of some complications; however, the observed complications rates were in line with published literature [20]. Nonetheless, the low occurrence of complications did result in a failure to assess the costs of complications using a bottom-up approach. As noted above, this limitation was addressed through use of locally published charges for hospitalization for a surgical gynecological procedure. The number of facilities where costing took place was also limited, and we interviewed providers rather than conducting time and motion surveys. However, the abortion providers performed MVA services on a daily or weekly basis and had done so for several years. Training costs were not included. This is important because although an NGO previously provided abortion training on behalf of government, that responsibility now falls on the public health sector. Training on abortion is not a part of routine nursing education in South Africa; instead it is offered as a kind of short course for willing health care professionals. Finally, some unit costs or clinical parameters could not be assessed at the study facilities and were drawn from published, South African literature.

Despite its introduction in the public sector in 2013, access to medication abortion is still not universal in South Africa. This analysis supports the scale-up of medication abortion alongside existing MVA services. The two procedures are similar in cost; this implies that scaling up medication abortion would not result in increased spending if service volumes were held constant. Further, given medication abortion’s slightly lower average cost per complete abortion, cost savings might be achieved due to women’s demonstrated preferences for the method. Reductions in the cost of mifepristone locally would contribute to greater savings. A change in South Africa’s policy on medication abortion to reflect the WHO’s guidance on follow-up visits after medication abortion and MVA [20] could further reduce the costs of medication abortion relative to MVA procedures.

Finally, having a choice of method has been shown to be important to women undergoing abortion [20,47]. Ensuring that services are offered in accordance with guidelines, and where possible, offering women a choice of method should be considered in order to improve women’s experiences of safe abortion services generally.

## Supporting information

S1 Form First visit interview guide. (PDF)

S2 Form First visit study nurse data collection form. (PDF)

S1 Workbook snapshot Model table of contents, decision tree, and dashboard where sensitivity analysis and final outcome calculations were done. (PDF)
