# Supplementary material for: The costs and cost effectiveness of providing first-trimester, medical and surgical safe abortion services in KwaZulu-Natal Province, South Africa
Source: PLoS One. 2017 Apr 3;12(4):e0174615. doi: 10.1371/journal.pone.0174615 (PMC5378341; doi:10.1371/journal.pone.0174615)
Supplement: S1 Form — (PDF) [file pone.0174615.s001.pdf]

**Client Interview Day One (CD1)**

| Today's Date | | | | | |
|---|---|---|---|---|---|
| d | d | m | m | y | r |

| Participant ID | | | | |
|---|---|---|---|---|
| | | | | |

**Facility ID**

| | | |
|---|---|---|
| | | |

*Instructions: (Study staff) Use this form to interview the woman after she has seen the health care provider and before she leaves the clinic on the same day that she was consented/enrolled into the study. For open responses, write the response in space provided. For Yes/No questions, tick the appropriate box.*

| Question | Response |
|---|---|
| 1. How old are you? | |
| 2. a. What is the highest grade in school that you completed? | a.❑ 1 = Grade 5 or less<br>❑ 2 = Grade 6-8<br>❑ 3 = Grade 9-10<br>❑ 4 = Grade 11<br>❑ 5 = Grade 12 – Matriculated |
| b. Are you currently in school? | b.❑ 0 = No<br>❑ 1 = Yes |
| c. Do you have a certificate, diploma, or degree beyond matriculation? | c.❑ 0 = No<br>❑ 1 = Yes, certificate or diploma.<br>*SPECIFY COURSE*:<br>____________________<br>❑ 2 = Yes, degree.<br>*SPECIFY DEGREE*:<br>____________________ |
| 3. What is your race? | ☐ African (1)<br>☐ White (2)<br>☐ Coloured (3)<br>☐ Indian (4)<br>☐ Other Asian (5) |

| Today's Date | | | | | |
|---|---|---|---|---|---|
| d | d | m | m | y | r |

| Participant ID | | | | |
|---|---|---|---|---|
| | | | | |

---

| | |
|---|---|
| 4. a. What is your marital status?<br><br>b. IF MARRIED, what type of marriage is it: | ☐ Married (1)<br>☐ Cohabiting (2)<br>☐ Divorced/separated (3)<br>☐ Single (4)<br>☐ Widowed (5)<br><br>☐ Legal (1)<br>☐ Traditional (2) |
| 5. How would you describe your employment situation? | ❑ 1= Unemployed looking for work<br>❑ 2 = Unemployed not looking for work<br>❑ 3 = Self-employed – working full-time<br>❑ 4 = Self-employed – working from time to time<br>❑ 5 = Employed, part-time<br>❑ 6 = Employed, full-time<br>❑ 7 = Unable to work<br>❑ 8 = Retired<br>❑ 9 = Other *SPECIFY*:<br>______________________________ |
| 6. Do you, or does anyone in your household, currently receive any kind of government grant?<br><br>*IF ANY:* What kinds of grants do you (*or do they*) receive?*TICK ALL THAT ARE MENTIONED* | ❑ 0 = No →*GO TO Q7*<br>❑ 1 = Yes<br><br>❑ 1 = Child grant<br>❑ 2 = HIV grant<br>❑ 3 = Pension grant<br>❑ 4 = Disability grant<br>❑ 5 = Other *SPECIFY*: ____________ |
| 7. What has been your primary source of income (or money) in the last 12 months?<br>*READ OPTIONS; CHOOSE ONE* | ❑ 0 = None<br>❑ 1 = Family<br>❑ 2 = Employment<br>❑ 3 = Spouse, boyfriend, girlfriend<br>❑ 4 = Grant *(CAN BE OWN GRANT OR CHILD'S GRANT)*<br>❑ 5 = Other *SPECIFY*: ____________ |
| 8. Are you currently caring financially for any dependents?<br>*IF YES:* Are your dependents children, adults, or both? | ❑ 0 = No<br>❑ 1 = Yes, children only<br>❑ 2 = Yes, adults only<br>❑ 3 = Yes, both children and adults |
| 9. Do the people in your household go without | ❑ 1 = Often<br>❑ 2 = Sometimes |

| Today's Date | | | | | |
|---|---|---|---|---|---|
| d | d | m | m | y | r |

| Participant ID | | | | |
|---|---|---|---|---|
| | | | | |

| | |
|---|---|
| food often, sometimes, seldom, or never? | ❑ 3 = Seldom<br>❑ 4 = Never |
| 10. a. Are you losing any income while you're at the clinic/hospital today?<br><br>b. IF YES, How much? | ☐ Yes (1)<br>☐ No (0)<br><br>How much: R _____ . ___ |
| 11. Do you have to pay for childcare while you are here?<br><br>IF YES, How much? | ☐ Yes (1)<br>☐ No (0)<br><br>How much: R _____ . ___ |
| 12. a. How much does it cost for you to come to the clinic/hospital from your home?<br><br>b. And how long does it take to come to the clinic/hospital from your house? | R ____. ___<br><br>____ ____ : ____ ____<br>H H M M |
| 13. What type of housing do you live in? | ☐ House (1)<br>☐ Flat (2)<br>☐ Cottage (behind another house) (3)<br>☐ Shack (4)<br>☐ Student Residence (5)<br>☐ Other (6)<br>If other, specify: |
| 14. Where is (Response to 13) located? | ☐ On the premises where you work(1)<br>☐ In the city (2)<br>☐ In an informal settlement(3)<br>☐ In a rural area (4)<br>☐ Other (5) |
| 15. How many rooms are there where you currently stay, excluding bathrooms, halls and passages? | |
| 16. Including yourself, how many adults and/or children live in that place? Please include anyone who stayed there at least 15 out of the last 30 days. | Total number of adults or children <u>> 16 years old:</u><br><br>Total number of children < 16 years old: |
| 17. What is the most often used source of <u>drinking</u> water at the place where you stay? | ❑ 1 = Piped – in the home<br>❑ 2 = Piped – tap in own yard/garden<br>❑ 3 = Piped - public tap/kiosk (free)<br>❑ 4 = Piped - public tap/kiosk (paid for)<br>❑ 5 = Other water source, |

| Today's Date | | | | | |
|---|---|---|---|---|---|
| d | d | m | m | y | r |

| Participant ID | | | | |
|---|---|---|---|---|
| | | | | |

| | |
|---|---|
| 18. What kind of toilet do you have at the place where you stay? | ❑ 1 = Flush toilet<br>❑ 2 = Improved pit latrine with ventilation (vip)<br>❑ 3 = Other pit latrine<br>❑ 4 = Bucket toilet<br>❑ 5 = Chemical toilet<br>❑ 6 = None |
| 19. Is that place connected to an electricity supply? | ❑ 0 = No<br>❑ 1 = Yes |
| 20. Do you have a television there? | ❑ 0 = No<br>❑ 1 = Yes |
| 21. Do you have a radio there? | ❑ 0 = No<br>❑ 1 = Yes |
| 22. How many times have you been pregnant and what were the results of each pregnancy? | Provider number of each:<br><br>______ Normal vaginal birth<br>______ Cesarean<br>______ Miscarriage (spontaneous abortion)<br>______ TOP<br>______ Ectopic pregnancy<br><br>______ Total |
| 23. How many live births have you had? | |
| 24. a. Have you ever used a family planning method?<br><br>b. IF YES, What methods have you used? | ☐ Yes (1)<br>☐ No (0)<br><br>List method(s): |
| 25. Before coming for this TOP, **when** did you first suspect that you were pregnant? | D D M M Y Y |

| Today's Date | | | | | |
|---|---|---|---|---|---|
| d | d | m | m | y | r |

| Participant ID | | | | |
|---|---|---|---|---|
| | | | | |

| | |
|---|---|
| 26. Why did you suspect that you were pregnant? | RESPONSE: |
| a. Did you have a positive pregnancy test before today? | ☐ Yes (1)<br>☐ No (0) |
| IF YES FOR 16A,<br>a1. When? | D D M M Y Y |
| a2. Where? | ☐ At home (1)<br>☐ At a clinic (2) |
| a3. How much did it cost? | R______ . ____ |
| 27. When did you decide to have a TOP? | |
| 28. What are your reasons for seeking a TOP? | OPEN RESPONSE: |
| 29. a. After deciding to have a TOP, did you go to a clinic immediately, or did you delay for some time? | ☐ Went immediately (1)<br>☐ Delayed (2) |
| b. IF DELAYED, For how long did you delay going to a clinic to ask for TOP? | How long delayed: |
| c. IF DELAYED, Why did you delay going to the clinic? | Why delayed: |

| Today's Date | | | | | |
|---|---|---|---|---|---|
| d | d | m | m | y | r |

| Participant ID | | | | |
|---|---|---|---|---|
| | | | | |

| | |
|---|---|
| 30. Before going to a clinic for a TOP, how did you get information on TOP? | |
| 31. a. Is this clinic/hospital the first place you went when you decided to have a TOP? | ☐ Yes (1)<br>☐ No (0) |
| b. When did you first come here/go there? | D D M M Y Y |
| c. IF NO FOR 21A, What was the first facility you went to? | List first facility: |
| d. What happened when you were there? What did they do? | OPEN RESPONSE: |
| 32. Why did you come to this facility for a TOP? | |
| 33. a. Before coming to this facility had you ever heard of medication abortion, or TOP by taking pills? | ☐ Yes (1)<br>☐ No (0) |
| b. IF YES, Did you know that medication abortion was available at this facility before you came here? | ☐ Yes (1)<br>☐ No (0) |
| 34. We know that some women in South Africa try to do TOP at home using herbs or tablets or drinking special mixes. Some women also try to do it outside of a clinic or hospital by calling special phone numbers that advertise TOPs. Before coming to this clinic/hospital, | ☐ Yes (1)<br>☐ No (0)<br>☐ IF YES, OPEN RESPONSE: |

| Today's Date | | | | | |
|---|---|---|---|---|---|
| d | d | m | m | y | r |

| Participant ID | | | | |
|---|---|---|---|---|
| | | | | |

| | |
|---|---|
| did you try any other methods to end this pregnancy?<br>IF YES, What did you try? | |
| 35. When you met with the doctor/nurse today, did you choose to have medication abortion or surgical abortion? | ☐ Medication abortion (1)<br>☐ Surgical abortion (2) |
| 36. Why did you choose that type of TOP? | |
| 37. Why did you not choose [MVA/MA] (SEE RESPONSE IN 36) | |
| 38. a. IF SHE CHOSE MEDICATION ABORTION, Did you receive mifepristone today?<br>b. And did you receive misoprostol tablets to take home?<br>c. And did you receive other pain medication? | ☐ Yes (1)<br>☐ No (0)<br>☐ Yes (1)<br>☐ No (0)<br>☐ Yes (1)<br>☐ No (0) |
| 39. a. IF SHE CHOSE SURGICAL ABORTION, When is your appointment for a surgical TOP? | Date/time: |
| 40. Were you using a contraceptive method when you became pregnant this time? IF YES, which one? | |
| 41. Were you told about contraception in your visit today? | |
| 42. Were you asked which contraceptive method you were using? | |

| Today's Date | | | | | |
|---|---|---|---|---|---|
| d | d | m | m | y | r |

| Participant ID | | | | |
|---|---|---|---|---|
| | | | | |

| | |
|---|---|
| 43. Were you given a contraceptive method today? | ☐ Yes (1)<br>☐ No (0) |
| a. IF NO, Would you have liked a contraceptive method? Why or why not? | a. |
| b. IF YES, What method did you receive? | b. |

44. **Follow-up interview date:** __________________ OR (circle) Not applicable

| | |
|---|---|
| ***Initials and date:*** | |

| |
|---|
| INTERVIEWER NOTES: |
