# Supplementary material for: The costs and cost effectiveness of providing first-trimester, medical and surgical safe abortion services in KwaZulu-Natal Province, South Africa
Source: PLoS One. 2017 Apr 3;12(4):e0174615. doi: 10.1371/journal.pone.0174615 (PMC5378341; doi:10.1371/journal.pone.0174615)
Supplement: S2 Form — (PDF) [file pone.0174615.s002.pdf]

Medication Abortion Screening Checklist (MASP)

Today's Date					
d	d	m	m	y	r

Participant ID				

Facility ID

Provider Initials

Instructions: (PROVIDER) Use this form to assess the woman's eligibility for medication abortion. For open responses, write the response in space provided. For Yes/No questions, tick the appropriate box.

I. MEDICATION ABORTION ELIGIBILITY AND PROCEDURE CHOICE

Question	Response	
1. Positive urine pregnancy test or other confirmation of pregnancy?	☐ Yes (1)	☐ No (0)
2. a. Gestational age ≤ 84 days (12 weeks)?	☐ Yes (1)	☐ No (0)
b. Gestational age ≤ 63 days (9 weeks)?	☐ Yes (1)	☐ No (0)
c. If yes, was this based on (tick all that apply)<br>i. LMP? *Date of LMP:*<br>_____dd______mm_____yy	☐ Yes (1)<br>COMPLETE DATE	☐ No (0)
ii. Provider's clinical assessment?	☐ Yes (1)	☐ No (0)
iii. Provider's ultrasound assessment?<br>*BPD:* ☐☐☐ mm<br>*CRL:* ☐☐☐ mm	☐ Yes (1)<br>COMPLETE MM.	☐ No (0)
3. Final gestational age assessment:	_______wks ______dys	
**IF NOT PREGNANT OR ABOVE 63 DAYS OF GESTATIONAL AGE, STOP HERE. IF 63 DAYS OR LESS, PROCEED.**		
4. Does she live or work within 1 hour of an emergency medical facility?	☐ Yes (1)	☐ No (0)
5. Is she willing to come for at least one follow-up visit?	☐ Yes (1)	☐ No (0)
6. a. Are there any contraindications to medication abortion?<br>b. IF YES, please specify:	☐ Yes (1)	☐ No (0)
If all of the answers to Questions 1 to 6 appear in shaded sections, the woman is eligible for medication abortion. Continue to complete this form.<br>*7.* **Is the patient eligible for medication abortion?**	☐ **Yes (1)**	☐ No (0)

Today's Date					
d	d	m	m	y	r

Participant ID				

If 7 is YES, provide information on medication abortion and ask the woman to choose either medication or surgical abortion.<br>8. Indicate her choice of procedures:	☐ Medical TOP (1)	☐ Surgical TOP (2)

II. BRIEF MEDICAL HISTORY

9. a. Does she take medication(s) on a daily basis?	☐ Yes (1)	☐ No (0)<br>SKIP B.
b. If yes, please note what medication(s) she takes (name of drug, dosage and frequency).		
10. a. Other comments/concerns (other medical conditions or history)<br><br>b. If yes, describe	☐ Yes (1)	☐ No (0)

Today's Date					
d	d	m	m	y	r

Participant ID				

III. COMPLETE THIS SECTION FOR MEDICAL ABORTION CLIENTS ONLY

If the woman has chosen medication abortion, administer mifepristone, and complete the following:

11. Time mifepristone taken by woman:	*____ h ____ (24 hr clock)*
12. Date mifepristone taken by woman:	*day ____/ month ____/ year ____*
13. Number of mifepristone tablets given:	
15. Lot number:	

Then provide her with 4 misoprostol tablets (200 μg each).

16. Number of misoprostol tablets given:	
17. Lot number(s):	

Provide the woman with analgesics and advise her to take for pain management (per guidelines established in training).

18. Pain medication given:	A. Type:<br>B. Dosage per tablet:<br>C. Number of tablets:

Then give her the Patient Information Sheet and explain to her that…

- ***She needs to take the misoprostol at home approximately 48 hours after taking mifepristone.***
- ***She may be nauseated, vomit or have diarrhea after taking misoprostol.***

Today's Date					
d	d	m	m	y	r

Participant ID				

- *She will probably also have abdominal pain or cramping stronger than menses.*
- *She will also have bleeding which may be heavier than she usually experiences with her menses and that all of these side effects are temporary.*
- *If she forgets to take misoprostol in 48 hours she should take it as soon as she remembers.*
- *Explain that she has been given a telephone number (in the consent form) to call in case of a medical emergency or if she has any questions or concerns during the study.*

IV. COMPLETE THISE SECTION FOR SURGICAL CLIENTS ONLY

19. Date booked for TOP	__ __ __ __ __ __<br>***D D M M Y Y***
