# Supplementary material for: The costs and cost effectiveness of providing first-trimester, medical and surgical safe abortion services in KwaZulu-Natal Province, South Africa
Source: PLoS One. 2017 Apr 3;12(4):e0174615. doi: 10.1371/journal.pone.0174615 (PMC5378341; doi:10.1371/journal.pone.0174615)
Supplement: S1 Workbook snapshot — (PDF) [file pone.0174615.s003.pdf]

Table of Contents - First-trimester abortion analysis

NB: Contents listing is hyperlinked to workbook tabs.

**Functional and analytical worksheets**	***Description***
Dashboard (cost summary and sensitivity analysis)	*Summarized listing of cost outcomes with levers/ranges for adjustments for sensitivity analysis*
Decision tree for cost-effectiveness calculations	*Clinical and service parameters X average costs per activity, including unscheduled visits*
Average cost per activity calculations	*Resource usage x unit costs per resource for abortion procedure activities (does not include unscheduled visits)*
Unscheduled visit cost calculations	*Resource usage x unit costs per resource for unscheduled visits*
Analysis parameters	*Listing of parameters (analysis year, discount rate, etc) which can be varied if desired*
**Research usage and clinical outcomes data**	***Source***
Clinical service parameters and outcomes	*Study database, provider interviews*
Service volume statistics, actual	*Study enrollment records*
Staff time - summary calculations	*Calculated based on detailed accounts (see below)*
Staff time - MA, detail	*Provider interviews*
Staff time - MVA, detail	*Provider interviews*
Supply usage	*Study database, provider interviews*
Equipment usage	*Study database, provider interviews*
Medication usage	*Study database, provider interviews*
Labs/diagnostics usage	*Study database, provider interviews*
**Appendices: Unit cost and other source information**	***Source***
Inflation, depreciation, exchange rates, etc.	*IMF, World Bank, Statistics South Africa, etc*
Personnel costs (per type and per minute)	*Calculated using DPSA tables and general accounting practice guidelines for South Africa*
Personnel salaries	*Department of Public Service Administration (DPSA), South Africa*
Supply costs	*National tenders and other sources*
Equipment costs	*National tenders and other sources*
Medication costs	*Master Procurement Catalog (national)*
Lab/diagnostic costs	*National Health Laboratory Service*
Hospitalization costs	*Uniform Patient Fee Schedule, South Africa*
Complication and completion rates	*Published literature*
Additional lists for model drop-down menus	
Full citations for all data sources	

Dashboard - 1st trimester

Back to TOC

Move the scroll bars below to make adjustment for uncertainty and sensitivity analysis.

Click Reset to return to observed parameter.

Uncertainty analysis	Measured value	UA Range	Adjustment		
Costs					
Staff time	--	± 25%	0%		Reset
			UA_staff_time		
Supply costs	--	± 25%	0%		Reset
			UA_supp_cost		
Equipment costs	--	± 25%	0%		Reset
			UA_equip_cost		
Hospitalization cost (staff & hotel)	4384	± 25%	0%		Reset
	305		*UA_hosp_cost*		
Hospitalization length (days)	1.0	.5-4.5	1		Reset

Sensitivity analysis	Measured value	SA Range	Adjusted value		
Cost inputs					
Mifepristone cost (200 mg)	227.51	(113.76-227.51)	0%		Reset
	Mife_cost		*SA_mife_cost*		
	227.51				
MVA lifespan (days)	30	7-30	0%		Reset
First trimester	30		*SA_MVA_asp*		
Second trimester					
Depreciation		3%, 5%		See analysis parameters page	
Service volume, % per service					
Service volume				Make sure defaults are set on Outcomes1T	
all sites	1129	455	2259	Swith all levers & change number on Tree.	
	Mean	*Min-Max*			
site 1	22.74	8-45	0%		Reset
	22.74				
site 2	39.36	17-78	0%		Reset
	38.36				
site 3	12.31	5-24	0%		Reset
	12.31				
% eligible for MA	78.5%	0%-100%	0.00%		Reset
	78.5%		*SA_Eliglble*		
% who choose MA	94.1%	0%-100%	0.00%		Reset
MA	94.1%		*SA_Chose*		
Clinical outcomes					
Completion rate	*completion_MA*				
MA	96.8%	95-98%	0.00%		Reset
	96.8%		*SA_complete_MA*		
MVA	100.0%	95-100%	0.00%		Reset
	100.0%		*SA_complete_MVA*		
Hospitalization/Complication (not incomplete)					
MA	0.4%	0-5.0%	0.00%		Reset
	hospitalization_MA		*SA_hosp_MA*		
	0.4%	NB: can't hospitalize more than the number of incompletes			
MVA	0.0%	0-5%	0%		Reset
	hospitalization_MVA		*SA_hosp_MVA*		
	0.0%	NB: can't hospitalize more than the number of incompletes			
Visits, follow-up					
Follow-up visit rate					
LTFU - MA	14.4%	0-100	0%		Reset
	14.4%		*SA_F.UP_LTFU_MA*		
Come for F.up:	85.6%				
MVA came for F.up	1	0-10%	(1 = 10%)		

Personnel - hospital only	*29.648*	*14%*			*39.216*	*0%*			*32.148*	*11%*		

	**MA - total average cost (ZAR)**			74%	**MVA - total average cost (ZAR)**			26%	**Both**			
	**Base estimate**		**Uncertainty analysis**		**Base estimate**		**Uncertainty analysis**		**Base estimate**		**Uncertainty analysis**	
	**Cost**	**% of total**	**Low**	**High**	**Cost**	**% of total**	**Low**	**High**	**Cost**	**% of total**	**Low**	**High**
Personnel	428.676	46.6%	322.02	535.33	564.297	56.3%	423.22	705.37	464.112	49.3%	348.46	579.76
Supplies	151.496	16.5%	113.64	189.36	277.115	27.7%	207.84	346.39	184.320	19.6%	138.25	230.39
Medication	249.584	27.1%	249.58	249.58	24.575	2.5%	24.58	24.58	190.791	20.3%	190.79	190.79
Equipment	76.205	8.3%	57.27	95.14	135.466	13.5%	101.60	169.33	91.689	9.7%	68.85	114.52
Laboratory	0.00	0.0%	0.00	0.00	0.00	0.0%	0.00	0.00	0.00	0.0%	0.00	0.00
"Hotel" cost (1 day)	13.712	1.5%	10.28	17.14	0.000	0.0%	0.00	0.00	10.129	1.1%	7.60	12.66
**Total**	**919.67**	**100.0%**	**752.79**	**1086.54**	**1001.45**	**100.0%**	**757**	**1246**	**941.041**	**100.0%**	**753.96**	**1128.12**
**Total cost**	**766 954**		**631 078**	**912 970**	**299 683**		**224 074**	**368 792**	**1062436**			

	**MA - total average cost (USD)**				**MVA - total average cost (USD)**							
	**Base estimate**		**Uncertainty analysis**		**Base estimate**		**Uncertainty analysis**		**Base estimate**		**Uncertainty analysis**	
	**Cost**	**% of total**	**Low**	**High**	**Cost**	**% of total**	**Low**	**High**	**Cost**	**% of total**	**Low**	**High**
Personnel	29.79	46.6%	22.38	37.20	39.22	56.3%	29.41	49.02	32.25	49.3%	24.22	40.29
Supplies	10.53	16.5%	7.90	13.16	19.26	27.7%	14.44	24.07	12.81	19.6%	9.61	16.01
Medication	17.34	27.1%	17.34	17.34	1.71	2.5%	1.71	1.71	13.26	20.3%	13.26	13.26
Equipment	5.30	8.3%	3.98	6.61	9.41	13.5%	7.06	11.77	6.37	9.7%	4.79	7.96
Laboratory	0.00	0.0%	0.00	0.00	0.00	0.0%	0.00	0.00	0.00	0.0%	0.00	0.00
"Hotel" cost (1 day)	0.95	1.5%	0.71	1.19	0.00	0.0%	0.00	0.00	0.70	1.1%	0.53	0.88
**Total**	**63.91**	**100.0%**	**52.32**	**75.51**	**69.60**	**100.0%**	**52.62**	**86.57**	**65.3973**	**100.0%**	**52.40**	**78.40**
**Total cost**	**53 299.20**		**43 856.54**	**63 446.52**	**20 826**		**15 571.94**	**25 629.07**	**73 833.6**			
Complete abortion cost	64.06				69.60							
Change in cost per comp. ab.	0.00%	0.00%			-1.4%	1.4%						
Comp. ab. cost after change	64.06				70.60							
ICER	2310.18											

Minutes of staff time required

	**MA**			**MVA**		
	**Base**	**Low**	**High**	**Base**	**Low**	**High**
Clerk	22.50	16.88	28.13	17.50	13.13	21.88
Social Worker	0.00	0.00	0.00	0.00	0.00	0.00
Staff Nurse	0.00	0.00	0.00	117.76	88.32	147.20
Professional Nurse	0.00	0.00	0.00	0.00	0.00	0.00
Nurse midwife	93.88	70.41	117.34	86.46	64.84	108.07
Medical Officer	0.00	0.00	0.00	0.00	0.00	0.00
Specialist	0.00	0.00	0.00	0.00	0.00	0.00
Ultrasound technician	14.97	11.23	18.72	14.51	10.88	18.13
Cleaner	1.81	1.35	2.26	12.64	9.48	15.80
Security Guard	0.00	0.00	0.00	0.00	0.00	0.00

Total requiring MVA:	23
Total requiring repeat MVA	0
Total hospitalized, MA:	3
Total hospitalized, MVA:	0

Decision Tree 1T

Back to TOC

Source: 8

KEY:

N	ZAR	%
MVA	MA	

RESET

Gestational age, eligibility	First visit - Choice (if applic.)	Procedure visit / Outcome	Unscheduled visit / Hospitalized?	Follow-up?	Total cost (ZAR)	Total cost (USD)	
Presented, eligible for 1st tri ab: 1129 | 100%							
> 9 wks, std of care MVA: 243 | 425 | 21.5%		MVA complete, no complication: 243 | 576 | 100%	No unscheduled visit: 243 | 0 | 100%	Follow-up, in person: 24.3 | 144 | 10%	27 840	1 935	Complete MVA
				LTFU: 218.7 | 0 | 90%	219 018	15 221	Complete MVA
			Unscheduled visit, no MVA: 0 | 91 | 0%	Follow-up, in person: 0 | 144 | 0%	-	-	Complete MVA
				LTFU: 0 | 0 | 100%	-	-	Complete MVA
		MVA complicated (inc. incomplete): 0 | 576 | 0%	No unscheduled visit: 0 | 0 | 0%	Follow-up, in person w/ MVA: 0 | 1001 | 0%	-	-	MVA, repeat MVA
				LTFU: 0 | 0 | 100%	-	-	MVA, needed repeat MVA but LTFU
			Unscheduled visit with MVA: 0 | 1001 | 100%	Follow-up, in person: 0 | 144 | 0%	-	-	MVA, repeat MVA
				LTFU: 0 | 0 | 100%	-	-	MVA, repeat MVA
			Hospitalized: 0 | 4384 | 0%	Follow-up, in person: 0 | 144 | 0%	-	-	Hospitalized for MVA
				LTFU: 0 | 0 | 100%	-	-	Hospitalized for MVA
		LTFU (before MVA): 0 | 0 | 0%			-	-	LTFU before MVA
≤ 9 wks, able to choose: 886 | 78.5%	Chose MVA: 52 | 425 | 5.9%	LTFU (before MVA): 0 | 0 | 0%			-	-	LTFU before MVA
		MVA complete, no complication: 52 | 576 | 100%	No unscheduled visit: 52 | 0 | 100.0%	Follow-up: 5.2 | 144 | 10.0%	5 957	**414**	Complete MVA
		33 w/FU + 19 LTFU, assumed complete		LTFU: 46.8 | 0 | 90.0%	46 868	3 257	Complete MVA
		100% completion rate	Unscheduled visit, no MVA: 0 | 91 | 0.0%	Follow-up: 0 | 144 | 0.0%	-	-	Complete MVA
				LTFU: 0 | 0 | 100.0%	-	-	Complete MVA
		MVA complicated (inc. incomplete): 0 | 576 | 0%	No unscheduled visit: 0 | 0 | 0.0%	Follow-up w/ MVA: 0 | 1001 | 0.0%	-	-	MVA, repeat MVA
				LTFU: 0 | 0 | 100.0%	-	-	MVA, needed repeat MVA but LTFU
			Unscheduled visit w/MVA: 0 | 1001 | 100.0%	Follow-up: 0 | 144 | 0.0%	-	-	MVA, repeat MVA
				LTFU: 0 | 0 | 100.0%	-	-	MVA, repeat MVA
			Hospitalized: 0 | 4384 | 0.0%	Follow-up: 0 | 144 | 0.0%	-	-	Hospitalized for MVA
				LTFU: 0 | 0 | 100.0%	-	-	Hospitalized for MVA

Chose MA: 834 | 770 | 94.1%

- LTFU, assumed complete, no follow-up, no unscheduled visit: 120 | 0 | 14.4% → 92 419 | 6 423 | LTFU MA case
- Complete, no complication: 691 | 0 | 82.9%
  - *1 visit each* — Unscheduled visit: 5 | 91 | 0.7%
    - Follow-up, in person: 3 | 144 | 60.0% → 3 017 | 210 | Complete MA
    - Follow-up, telephonic: 0 | 51 | 0.0% → - | - | Complete MA
    - Follow-up, unknown (assume in-person): 2 | 144 | 40.0% → 2 011 | 140 | Complete MA
  - No unscheduled visit: 686 | 0.00 | 99.3%
    - Follow-up, in person: 555 | 144 | 80.9% → 507 472 | 35 267 | Complete MA
    - Follow-up, telephonic: 104 | 51 | 15.2% → 85 426 | 5 937 | Complete MA
    - Follow-up, unknown (assume telephonic): 27 | 51 | 3.9% → 22 178 | 1 541 | Complete MA
- Complicated (inc. incomplete): 23 | 0 | 2.8%
  - *1 had 4, 2 had 2, rest 1* — Unscheduled visit (1), MVA: 14.00 | 1097 | 0.6086957
    - Follow-up, in person: 13 | 144 | 92.9% → 26 148 | 1 817 | MA + MVA (no hosp)
    - Follow-up, telephonic: 0 | 51 | 0.0% → - | - | MA + MVA (no hosp)
    - Follow-up, unknown (assume in-person): 1 | 144 | 7.1% → 2 011 | 140 | MA + MVA (no hosp)
  - Unscheduled visit (2), MVA: 3.00 | 1188 | 13.0%
    - Follow-up, in person: 3 | 144 | 100.0% → 6 308 | 438 | MA + MVA (no hosp)
  - Unscheduled visit (4), MVA: 1.00 | 1371 | 4.3%
    - Follow-up, in person: 1 | 144 | 100.0% → 2 285 | 159 | MA + MVA (no hosp)
  - *All hospitalized* — No unscheduled visit, MVA with hospitalization: 3.00 | 4384 | 13.0%
    - Follow-up, in person: 2 | 144 | 66.7% → 10 597 | 736 | MA+Hospitalized for MVA
    - Follow-up, telephonic: 1 | 51 | 33.3% → 5 205 | 362 | MA+Hospitalized for MVA
  - *1 UV was a call* — Unscheduled IN-PERSON visit, ongoing pregnancy (W/ MVA OFFSITE): 1.00 | 91 | 4.35%
    - Follow-up, in person: 1 | 144 | 0.0% → 1 006 | 70 | MA, ongoing, MVA done elsewhere
    - Follow-up, telephonic: 0 | 51 | 100.0% → - | - | MA, ongoing, MVA done elsewhere
  - *1 UV was a call* — Unscheduled TELEPHONIC visit, ongoing pregnancy (W/ MVA OFFSITE): 1.00 | 51 | 4.35%
    - Follow-up, in person: 0 | 144 | 0.0% → - | - | MA, ongoing, MVA done elsewhere
    - Follow-up, telephonic: 1 | 51 | 0.0% → 873 | 61 | MA, ongoing, MVA done elsewhere
    - LTFU: 0 | 0 | 100.0% → - | - | MA, ongoing, MVA done elsewhere

96.8% completion rate

3.2% Incomplete rate

0.4% Hospitalization rate

	N	%	Adj. N	Adj %		
Follow-up (MA)	0	--	0			
In-person	578	69.3%	581	69.7%	*	F.up_person
Telephonic	106	12.7%	133	15.9%		F.up_tele
F.up, but unknowr	30	3.6%	0	0.0%	**	
LTFU	120	14.4%	120	14.4%		F.up_LTFU

*Includes 1 SAE/ongonig pregnancy, that was classified incorrectly as in-person.

**If had an unscheduled visit, f.up = in-person. If no UV, = telephonic.

Complicated cases - MA				
		No SA	w/SA	
UV1	14	60.9%	70%	0.05
UV2	3	13.0%	15%	0.01
UV4	1	4.35%	5%	0.00
Hosp	3	13.0%	--	35.37
UV-Per,	1	4.35%	5%	0.00
UV-Tel, (	1	4.35%	5%	0.00

Average cost per procedure (USD)

Note

Complete MVA, no complication	#	Total
70.60	295	20826

MVA, complicated	#	
--	0	0

MVA all	#	
70.60	295	20826

Average - all	#	Total
65.656	1129	74126
**Average - complicated**		
164.47	23	3783
**Average - not complicated**		5.1%
63.60	1106	70343
**Cost per complete abortion**		95.3%
65.77	1127	74126

Complete MA, no complication	#	Total
61.06	811	49516
	97.2%	92.9%

MA complicated	#	
164.47	23	3783
	2.8%	7.1%

MA all	#	
63.91	834	53299

Cost per complete abortion		
64.06	832	53299
